# Cochlear dysfunction is associated with styrene exposure in humans

**DOI:** 10.1371/journal.pone.0227978

**Published:** 2020-01-21

**Authors:** Mariola Sliwinska-Kowalska, Adrian Fuente, Ewa Zamyslowska-Szmytke

**Affiliations:** 1 Department of Audiology and Phoniatrics, Nofer Institute of Occupational Medicine, Lodz, Poland; 2 Centre de recherche de l’Institut universitaire de gériatrie de Montréal, Québec, Canada; 3 École d’orthophonie et d’audiologie, Faculté de médecine, Université de Montréal, Québec, Canada; Universidad de Chile, CHILE

## Abstract

**Aim:**

Occupational exposure to styrene has been shown to be associated with an increased probability of developing hearing loss. However, the sites of lesions in the auditory system in humans remain unknown. The aim of this study was to investigate the possible adverse effects of styrene exposure on the cochlea of human subjects.

**Design:**

The hearing function of 98 styrene-exposed male workers from the glass fibre-reinforced plastics industry (mean concentration of 55 mg/m^3^) was evaluated bilaterally using pure-tone audiometry (1000–16000 Hz), distortion product otoacoustic emissions (DPOAEs), and auditory brainstem response (ABR). The results were compared to a group of 111 male workers exposed to noise (above 85 dBA) and 70 male white-collar workers exposed to neither noise nor solvents. Age and noise exposure levels were accounted for as confounding variables in all statistical models.

**Results:**

Styrene exposure was significantly associated with poorer pure-tone thresholds (1–8 kHz), lower DPOAE amplitudes (5–6 kHz), and shorter wave V latencies in both ears compared to control-group subjects. Similar results were found among noise-exposed subjects. A further analysis with wave V latency showed that styrene-exposed subjects showed significantly shorter latencies than expected according to normative data. These results suggest that occupational exposure to styrene at moderate concentrations is associated with cochlear dysfunction, at least at high frequencies. DPOAEs may be considered a valuable diagnostic tool in hearing conservation programs in workers exposed to styrene.

## Introduction

Styrene is an aromatic solvent that is widely used as a precursor for polystyrene plastics. Pure styrene is a colourless, easily evaporating liquid with a characteristic sweetish odour and is partially soluble in water. In the manufacturing industry, it is mainly used in the production of polyester laminates and plastics, synthetic rubber and insulating materials (such as polyurethane foam), and in the glass fibre-reinforced plastic product industry (e.g., yachts, lavatory pans and washbasins). The highest occupational exposure to styrene occurs when laminating large items such as boats. Styrene is absorbed through the respiratory airways and the skin. Its metabolites are mainly mandelic acid (MA) and phenylglyoxylic acid (PGA). The occupational exposure limit (OEL) for styrene in the U.S. is 426 mg/ m^3^ (100 parts per million (PPM)) as an 8-hour time-weighted average (TWA), according to the Occupational Safety and Health Administration (OSHA), and 87 mg/m^3^ (20 PPM) as an 8-hour TWA, according to the American Conference of Governmental Industrial Hygienists (ACGIH). In Poland, the OEL for styrene is 50 mg/m^3^ (12 PPM) as an 8-hour TWA.

Chronic exposure to styrene has been associated with neurotoxicity [1,2], neuroendocrine alterations [3,4], nephrotoxicity [5], hepatotoxicity [6], acute myeloid leukemia [7], among other health problems. In addition, it has been suggested that styrene may adversely affect the hearing and balance systems [8,9].

Pryor et al. [10] were the first to report ototoxicity induced by styrene in an animal model. The adverse effect of styrene on human hearing was then initially reported by Muijser et al. [11], who found a statistically significant difference in hearing thresholds between workers directly exposed to styrene (at a mean concentration of 138 mg/m^3^) and workers indirectly exposed to styrene (at 61 mg/m^3^). However, Möller et al. [12], who examined a group of workers exposed to styrene at concentrations considerably lower than the Swedish OEL (i.e., 119 mg/m^3^ in 1990), did not find any significant hearing losses in the styrene-exposed group compared to non-exposed control subjects. Similarly, Calabrese et al. [13] found no hearing impairment in workers exposed to styrene concentrations of approximately 229 mg/m^3^. Some limitations, however, can be identified in these studies [12,13]. First, both studies [12,13] included a limited number of workers (18 and 20 subjects, respectively). Secondly, Calabrese et al. [13] did not use a control group; thus, the hearing test results for the styrene-exposed subjects were not compared to those of unexposed subjects. In addition, Calabrese et al. [13] did not provide details about the hearing tests utilised.

Three different studies have found poorer hearing thresholds in workers exposed to styrene [14–16], than in unexposed workers. Morata et al. [15], found that styrene-exposed workers, despite being exposed to low airborne styrene concentrations (below 5 ppm; 12–16 mg/m3), showed poorer audiometric pure-tone thresholds for the frequencies 2–6 kHz in comparison to non-exposed control subjects. Mandelic acid concentration in urine and the odds ratio for hearing loss were significantly correlated [15]. Morioka et al. [14], showed that styrene exposure (mean exposure of approximately 229 mg/m^3^) was correlated with a poorer upper frequency limit of hearing [17]. In our previous study [16], we showed that styrene exposure at a mean concentration of 61.8 mg/m^3^ was significantly associated with a 5.2-fold increased odds ratio of hearing loss; this value rose to 13.1-fold for the combined exposure to styrene and toluene. Mean hearing thresholds adjusted for age, gender and noise exposure were significantly poorer in the styrene-exposed group than in the non-exposed control group at all standard frequencies tested (1–8 kHz).

The results of the studies carried out in populations exposed to both noise and styrene are equivocal. Sass-Kortsak et al. [18] did not provide further evidence of styrene-induced hearing loss in 299 workers exposed to styrene and noise at fibre-reinforced plastic manufacturing factories. Noise exposure (Leq 87.2±6.5 dBA) and styrene exposure (concentration 73.5±88.6 mg/m^3^) were highly correlated and the effect of noise was shown to be predominant. In our previous study investigating the odds ratio for hearing loss in styrene-only, noise-only, and styrene-and-noise-exposed subjects, we suggested an interaction effect for co-exposure [16]. The probability of developing hearing loss increased 3.3-fold in noise-only exposed workers, 5.2-fold in styrene-only exposed workers, 10.9-fold in noise-and styrene co-exposed subjects and over 21.5-fold in noise, styrene and toluene co-exposed individuals.

Despite the evidence of an increased probability of developing hearing loss due to styrene exposure, a research gap remains concerning the auditory structures mainly affected by styrene exposure in humans. Potentially, styrene may adversely affect the central auditory system and the cochlea. This is because animal studies have demonstrated a clear adverse effect of styrene on the cochlea (e.g., [19, 20]). Outer hair cells (OHCs) and supporting cells in the organ of Corti have been shown to be initially damaged in rats exposed to a styrene concentration of 1000 ppm (4586mg/m^3^; [19]). Note, however, that the styrene concentrations used in animal studies are much higher than the levels normally found in the workplace. In addition, some human studies have suggested a central, rather than peripheral, auditory dysfunction associated with styrene exposure. Möller et al. [12] found no hearing loss in 18 workers with long-term exposure at low levels of styrene, as assessed by pure-tone audiometry. However, seven out of eighteen subjects obtained abnormal results for distorted speech and/or cortical response audiometry (CRA). Another study showed that styrene-exposed subjects had significantly poorer results in temporal processing tasks compared to non-exposed subjects [21]. In addition, Johnson et al. [22] found a significantly poorer performance on the interrupted speech test in styrene-exposed subjects than in noise-only and non-exposed subjects.

Considering the cochlear dysfunction observed in animals exposed to styrene and the adverse central auditory effect observed in human subjects exposed to styrene, it may be hypothesised that styrene induces both peripheral and central auditory dysfunction in humans. However, none of the studies conducted so far in human subjects have demonstrated cochlear impairment associated with styrene exposure.

The aim of this research study was to investigate the possible adverse effects of styrene exposure on the cochlea of human subjects exposed to this solvent in the workplace. For this aim, a comprehensive audiological test battery was used.

## Materials and methods

Ethical approval was obtained from the Ethics Committee of the Nofer Institute of Occupational Health, Lodz, Poland, prior to the commencement of the study. All participants who agreed to take part in the study signed an informed consent form.

### Subjects

The study sample included 98 styrene-exposed male workers from a plastic factory, 111 noise-exposed male workers from a dockyard and metal factory, and 70 white-collar male workers exposed to neither noise nor organic solvents (control group). These groups were selected from a larger sample of workers described elsewhere [16]. Exclusion criteria included the duration of employment with styrene/noise exposure being less than 6 months, abnormal otoscopy, an abnormal tympanogram (results different than type A), a history of ear diseases, ear surgery, or severe head trauma, and a family history of hearing loss. The group of noise-exposed subjects was older on average than the styrene-exposed and control groups. Table 1 provides characteristics such as exposure levels for the three groups of workers. Workers exposed to noise levels above 85 dB Leq (40 hours/week) were provided with hearing protection of different types depending on noise levels and personal preferences. The use of hearing protection based on factory records was considered to be adequate. Styrene-exposed workers were provided with face-masks or half-masks based on their specific duties and levels of exposure. Similarly, special gloves were provided to be used when skin contact with solvents was supposed to occur. However, the use of personal protective equipment against solvents was not regularly controlled although most workers reported to use it when needed.

**Table 1:** Characteristics of the groups of workers.

Variable		Styrene-exposed group	Noise-exposed group	Non-exposed control group
Number of subjects		98	111	70
Age [years]	Mean (SD)	34.1 (8.0)	39.2 (9.6)	34.3 (8.1)
	Range	20–58	22–55	21–56
Current styrene exposure [mg/m^3^]^1^	Mean (SD)	57.9 (31.4)	-	-
	Range	1.6–147.9	-	-
% overexposed		40.3		
Lifetime styrene exposure [mg/m^3^]	Mean (SD)	635.5 (703.7)	-	-
	Range	3.0–3807.0	-	-
Current exposure index ^2^	Mean (SD)	4.4 (2.2)	-	-
	Range	0.1–7.1	-	-
- % overexposed		94		
Mean lifetime noise exposure [dBA]^3^	Mean (SD)	79.4 (2.9)	92.4 (4.1)	76.1 (2.7)
	Range	74.0–85.0	85.1–100.1	75–85
Total lifetime noise exposure [dBA]	Mean (SD)	123.2 (4.1)	138.4 (4.7)	119.5 (6.2)
	Range	109.6–130.9	126.4–149.0	106.6–133.1

^1^ Admissible Occupational Exposure Limit for styrene in Poland is 50 mg/m^3^ (= 12ppm).

^2^ This value accounts for all solvents present in the workplace, i.e. styrene and acetone (or dichloromethane); the normative value is 1.

^3^ Noise exposure level normalised to a nominal 8h working day averaged over the total time of employment.

Polish Occupational Exposure Limit Value is L_EX,8h_ = 85 dBA, with 3 dB exchange rate.

### Sample selection procedures

#### Medical and occupational history questionnaire

This questionnaire was based on the noisechem questionnaire [23] and included detailed inquiries about present and previous exposure to solvents and noise, medical history, and non-occupational exposure to ototoxic agents. The medical history questions inquired about signs and symptoms relating to the auditory system, past middle-ear diseases and surgery, hereditary disorders, chronic systemic diseases, cholesterol level and hypertension, head trauma and current and past medications containing potential ototoxic agents. The occupational history questions addressed changes in duties performed, the place and conditions of work, the regular use of individual protection (type, availability and actual use), and a detailed analysis of exposure to noxious agents in former places of work. Questions on the history of non-occupational hearing risks included past noise exposure during military service or leisure activities.

#### Solvent exposure assessment

The evaluation of current occupational exposure to organic solvents was based on an assessment of the concentrations of all toxic compounds in the work environment, using an individual dosimetry method. In addition, the protocols of environmental inspections performed by local occupational safety agencies over the last 15 years were inspected to investigate exposure in former places of work.

The current airborne styrene concentrations ranged from 1.6 to 147.9 mg/m^3^ (Polish OEL -50 mg/m^3^ ~ 12 ppm.). In addition to the styrene used in the direct production line, two other organic solvents, acetone (detected concentrations from 1 to 200.8 mg/m^3^, OEL in Poland 600 mg/m^3^) and dichloromethane (detected concentrations from 1 to 102.17 mg/m^3^, OEL in Poland 20 mg/m^3^), were used in the factory. An exposure index for the mixture of organic solvents was calculated, including airborne concentrations for the aforementioned solvents (i.e., styrene, acetone and dichloromethane). The exposure index is a sum of the fractions (measured airborne concentration of a given chemical divided by its OEL value) for all mixture compounds. A value of 1 was set as the admissible level of exposure.

Since the employees were exposed to different solvent concentrations in different workplaces and during various employment periods, the average total working life exposure was calculated for each individual worker. The details of this procedure are given elsewhere [16]. Table 1 provides details about solvent exposure levels.

#### Noise exposure assessment

Subjects’ individual working life exposures to noise were evaluated based on collected work histories and exposure data in different working conditions (i.e., jobs). The records of mandatory periodic measurements made by employers over the last 20 years were analysed to obtain previous exposures to noise. Missing data were substituted with the best available information on the possible exposure parameters. Details about the procedures for the assessment of noise exposure in this group of workers are given elsewhere [16]. The mean lifetime equivalent noise levels in all cases were not above 85 dB(A) for both the styrene-exposed group and the control group. The mean levels ranged between 85.1 and 100.1 dB(A) in the noise-exposed group (see Table 1).

#### Audiological procedures

A hearing examination was performed at least 16 hours after the last exposure to noise/solvents in a soundproof booth that met the requirements of ISO 8253–1 [24]. The following procedures were carried out:

- Tympanometry was carried out using a Madsen Zodiac 901 middle-ear analyser. A probe signal of 226 Hz set to a continuous intensity of 85 dB SPL was used.
- Standard and ultra-high frequency pure-tone audiometry (air conduction 1–16 kHz, bone conduction 1–4 kHz) was carried out using an Interacoustics AC40 clinical audiometer. For standard frequencies, hearing thresholds higher than 25 dB HL were considered abnormal. For ultra-high frequency hearing thresholds, an ear with no measurable hearing thresholds at 12 and/or 16 kHz was noted.
- Otoacoustic emissions were carried out using an Otodynamics ILO96 to register, record and analyse distortion product otoacoustic emissions (DPOAEs). DPOAEs were registered in terms of the function of frequency (DP-gram). DP grams were recorded at 1.5, 2, 3, 4, 5 and 6 kHz, using 70 dB SPL equal levels of stimuli [25]. The frequency ratio f2/f1 was kept constant at 1.2. The measurement of DPOAEs utilised signal averaging until the noise level reached the lowest possible value. The analysed parameter was dB signal-to-noise ratio (SNR).
- The auditory brainstem response (ABR) was carried out using a Nicolet Biomedica Nicolet Spirit 2000 & Spirit 2000 Lite with Ag-AgC1 electrodes. The 10–20 system for electrode placement was used. The reference electrode was placed on the measured ear mastoid, the active electrode over the vertex and the ground electrode on the forehead. A 100 μs, 90 dB nHL click stimulus of alternating polarity was presented at a rate of 20 Hz. The analysed parameters were the latencies of waves I, III and V and the I-III, III-V and I-V inter-peak latencies (IPL).

### Data analysis

Data were analysed and plotted with R [26–28]. Data inspection revealed a small proportion of missing values in both the right ear data set (0.9%) and the left ear data set (0.033%). Values were imputed for 15 participants in the right ear data set and for 16 participants in the left ear data set. Since these missing values were randomly scattered among variables, an Expectation-Maximisation (EM) multiple imputation was conducted individually on each data set.

To gauge the effects of styrene exposure, hierarchical linear regression (HLR) analyses were conducted for both ears on all auditory outcomes. All HLR analyses were sequenced as follows: step 1 was always a one-predictor model including only age. In step 2, mean lifetime noise exposure was added. In the third and final model (step 3), group category was entered (i.e., styrene-exposed, noise-exposed, and control group). In step 2 (when entering mean lifetime noise exposure) and step 3 (when entering group category), the increase in the explained sum of squares was obtained, along with the corresponding *F*-values and *p*-values. Thus, the *p*-values observed in step 2 and step 3 provide a statistical measure of the impact of the sequentially entered variables on the prediction of a given outcome variable. A significant *p*-value in step 2 reveals a statistically relevant effect of mean lifetime noise exposure when controlling for age. A significant *p*-value in step 3 represents a statistically relevant effect of group category when controlling both for age and mean lifetime noise exposure. In other words, a statistically significant contribution of group category to the prediction of a given outcome variable basically sheds light on the effect of styrene exposure on the particular response variable observed. This is because both age and mean lifetime noise exposure were already present in the previous model (step 2). Also, since mean lifetime noise exposure is related with the criteria used to group the participants, the inclusion of group category in the final model mainly adds information relative to styrene exposure, which was not present in the two previous models. Given all this, when entering the group variable, the contrasts were set using the control group as reference. Thus, two contrasts were explored: control against styrene and control against noise. The reported results consider in detail all the analyses that revealed a significant change in prediction (α = 0.05) or a trend-wise change (p-value below 0.1) in step 3. Data were inspected for influential cases and seven participants were excluded based on consistently extreme studentised residuals values and/or high hat-values.

When adding group category improved the prediction, a non-parametric BCa bootstrap with 10,000 replications was conducted on the three-predictor model. This technique allows for a more accurate estimation of parameters (regression coefficients) by generating multiple random samples (with replacement) using the original observed sample. Parameters are calculated for each sample, and final regressors’ significance (based on non-zero confidence intervals) better captures the underlying effects. Among the many available methods to bootstrap regression coefficients, BCa was chosen because it is known to control bias and skewness [29, 30]. The rationale for using this technique was to further control between-group age differences, while providing a robust measure of significance for the observed predictors. In bootstrap analyses, a predictor is significant when its confidence interval (CI) is non-zero. Therefore, for a bootstrapped predictor to be deemed significant, the lower bound and the upper bound must both be either positive or negative. Reported 95% CIs will always present the lower bound on the left and the upper bound on the right. When relevant, adjusted *R*^2^s for the three-predictor models are also discussed and Cohen’s *f*^*2*^ is provided as an effect size measure for a given model. HLR analyses were conducted individually on the following response variables for both ears: average hearing thresholds across frequencies 1–8 kHz; DPOAE responses in SNR at 1.5 kHz, 2 kHz, 3 kHz, 4 kHz, 5 kHz, and 6 kHz; ABR wave V latency; and delta ABR-V. The latter was calculated based on the following procedure. The latency for ABR wave V obtained in each ear in every single subject at 90 dB nHL was plotted against the latency expected on each subject’s pure-tone thresholds between 4 and 6 kHz (dB SL) based on previously normative data [31]. For this, the dB SL at which click stimuli were presented in each subject was calculated by subtracting the average pure-tone threshold between 2–4 kHz from 90 dB nHL. Then, the wave V latency (in ms) expected at the resulting dB SL was subtracted from the latency obtained in the subject. The following formula from Prosser and Arslan [31] was used: Delta = Lp(90)–Ln(90-x). In this formula, Lp(90) is the actual latency value (in ms) for wave V (with a stimulus at 90 dB nHL) obtained in the subject. Ln(90-x) is the wave V latency value for a specific intensity level stimulus (90-x), as predicted from a normal intensity/latency function [31], where x represents the subject’s average pure-tone threshold for 2–4 kHz.

Finally, the number and percentage of subjects in each group (styrene-exposed, noise-exposed, and control) with no measurable response for the ultra-high frequency (12 and 16 kHz) hearing thresholds were calculated for each ear. A Chi-Square test was computed in order to explore the possible association between the categorical variables of exposure (styrene, noise, and control) and the absence of measurable responses for 12 and 16 kHz in each ear. Considering that the p-values for the Chi-Square test do not provide detailed information about the association between variables in tables that are larger than 2x2, the interpretation for this test was based on the analysis of Pearson’s residuals. Such residuals are deemed to be significant whenever exceeding the ±2.0 range [32]. Thus, a residual that is higher than 2.0 indicates that the number of cases in that cell is significantly larger than would be expected if the null hypothesis were true. Conversely, a residual that is less than 2.0 indicates that the number of cases in that cell is significantly smaller than would be expected if the null hypothesis were true.

## Results

### Pure-tone thresholds

**Fig 1:**
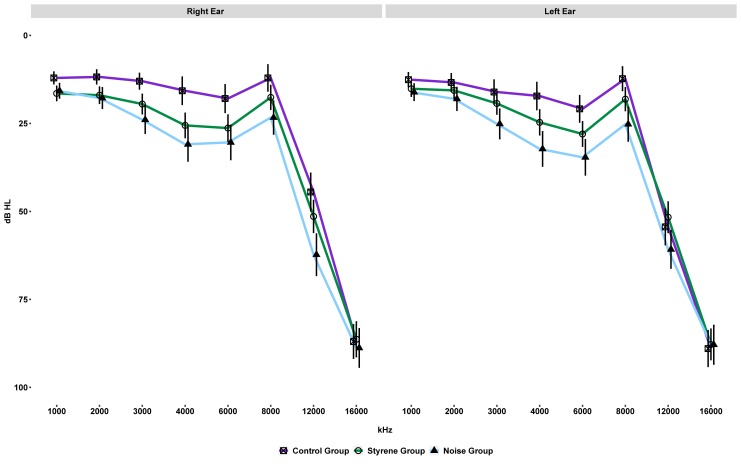
Mean and 95% confidence interval for pure-tone thresholds (1–16 kHz) for the right and left ears for each group of workers.

Fig 1 plots the means for pure-tone thresholds (1–16 kHz) of the three groups for the right and left ears (1–16 kHz).

HLR analysis revealed that both age (step 1) and mean lifetime noise exposure (step 2) significantly affect the prediction of the average hearing thresholds across frequencies (1–8 kHz) in both ears (see Table 2). A significant contribution of group category to the prediction of values for this auditory outcome was also observed. As for the right ear, this contribution translated into significant values when contrasting the two-predictor model in step 2 against the three-predictor model in step 3 (*F =* 7.64, *p <* 0.001). The adjusted *R*^2^ for the three-predictor model was 0.37 (*f*^*2*^ = 0.58). When bootstrapping coefficients, significance was observed for age (CI 0.6539, 0.9936) and the control/styrene contrast (CI 2.660, 8.8831). Bootstrapped coefficients for mean lifetime noise exposure and the control/noise contrast were not significant. Based on the coefficient of the regression (β), styrene-exposed workers are expected to present hearing thresholds around 5.9 dB HL higher (i.e. worse) than control-group workers in the right ear. Results for the left ear are very similar (see Table 2), with the predictors of models in steps 1 and 2 significantly affecting the outcome variable and with group category also statistically contributing to the prediction in step 3 (*F =* 3.81, *p =* 0.02, *R*^2^ = 0.38, *f*^*2*^ = 0.61). Bootstrapped coefficients were significant for age (CI 0.7009, 1.0153) and the control/styrene contrast (CI 1.081, 8.144). Based on the coefficient of the regression (β), styrene-exposed workers are expected to present hearing thresholds around 4.8 dB HL higher (i.e. worse) than control-group workers in the left ear.

**Table 2:** HLR results for the average of pure-tone thresholds across frequencies (1–8 kHz) for the right and left ears. Bootstrapped parameters (CI, bias and SE) are only reported when the increase in step 3 was found to be significant (< 0.05) or trend-wise (<0.1). All significant improvements in step 2 and/or step 3 are marked with **. Trend-wise improvements are marked with *.

**Right ear**						
	β	p	R^*2*^	CI	Bias	SE
**Step 1**						
Age	0.82	<0.001	0.33			
**Step 2**						
Age	0.79	<0.001	0.34**			
Mean lifetime noise exposure	0.20	0.01				
**Step 3**						
Age	0.81	<0.001	0.37**	0.6539, 0.9936	-0.001	0.086
Mean lifetime noise exposure	0.23	0.19		-01285, 0.6531	-0.004	0.19
Control/Styrene	5.94	<0.001		2.660, 8.8831	0.019	1.58
Control/Noise	1.83	0.59		-5.793, 8.896	0.085	3.69
**Left ear**						
	β	p	R^*2*^	CI	bias	se
**Step 1**						
Age	0.88	<0.001	0.36			
**Step 2**						
Age	0.86	<0.001	0.37**			
Mean lifetime noise exposure	0.17	0.03				
**Step 3**						
Age	0.85	<0.001	0.38**	0.7009, 1.0153	0.0000	0.07
Mean lifetime noise exposure	0.01	0.92		-0.4068, 0.4604	0.003	0.22
Control/Styrene	4.82	0.006		1.081, 8.144	3.11	1.79
Control/Noise	4.97	0.16		-3.900, 13.198	0.09	4.38

β: Estimated coefficient of the exploratory variables that indicates changes in the auditory outcome. Note that coefficients are not standardised.

**p<0.05

**Fig 2:**
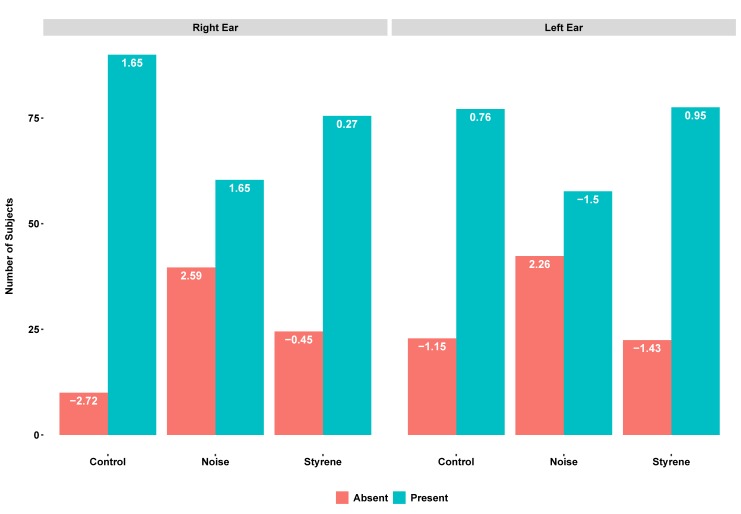
Pearson’s residuals (values are displayed in white on top of bars) for the Chi-Square test between group category and the absence/presence of measurable hearing threshold at 16 kHz. Values that are higher than 2.0 indicate that the number of cases in that cell is significantly larger than would be expected if the null hypothesis were true. Values that are less than 2.0 indicate that the number of cases in that cell is significantly smaller than would be expected if the null hypothesis were true. Y-axis represents the number of subjects in each category.

The results for the Chi-Square tests between the categorical variables of group category (styrene-exposed, noise-exposed, and control) and the absence/presence of measurable hearing threshold for the ultra-high frequencies (12 and 16 kHz) were 3.16 (p> .05) and 19.39 (p < .0001) for 12 and 16 kHz for the right ear, and 5.63 (p>.05) and 11.83 (p < .01) for 12 and 16 kHz for the left ear, respectively. Pearson’s residuals showed that the absence of a measurable threshold at 16 kHz was significantly low for the control group (less than -2.0) and significantly high for the noise-exposed group in the right and left ears (higher than 2.0). See Fig 2.

Fig 2 displays the Pearson’s residuals for the Chi-Square test between the categorical variable of group category (styrene-exposed, noise-exposed, and control) and the absence/presence of measurable hearing threshold at 16 kHz.

### Distortion product otoacoustic emissions

**Fig 3:**
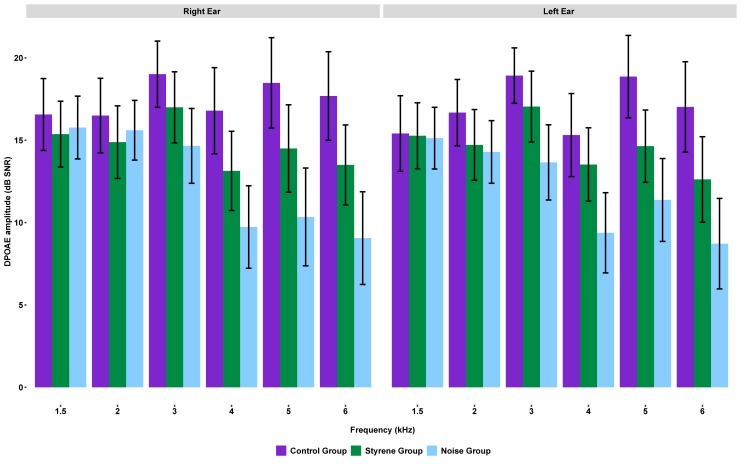
Mean amplitude (dB SNR) and 95% confidence interval for distortion product otoacoustic emissions across frequencies (1.5–6 kHz) for each group of workers for the right and left ears.

Fig 3 plots the mean amplitude in dB SNR for DPOAE from 1.5 to 6 kHz for the right and left ears across groups.

HLR analyses were conducted for DPOAEs at 1.5 kHz, 2 kHz, 3 kHz, 4 kHz, 5 kHz and 6 kHz in both ears. Results showed that for 1.5 kHz to 3 kHz, only age (step 1) significantly predicts the corresponding outcome variable in both ears (the only exception being results for the left ear at 3 kHz, which showed a significant effect of mean lifetime noise exposure (see Table 3). At 4 kHz, age (step 1) and mean lifetime noise exposure (step 2) significantly affect the prediction of this outcome in both ears. No significant effect of group category (step 3) was observed. At 5 kHz in the left ear, age, mean lifetime noise exposure and group category all significantly predict this outcome variable. Significant values when contrasting the two-predictor model in step 2 against the three-predictor model in step 3 were found (*F =* 3.92, *p =* 0.02, *R*^2^ = 0.29, *f*^*2*^ = 0.41). Significant bootstrapped coefficients for the three-predictor model were observed for age (CI -0.6884, -0.4575) and the control/styrene contrast (CI -6.864, -0.833). For the right ear (5 kHz), results were significant in steps 1 and 2 and trend-wise in step 3 (*F =* 2.53, *p =* 0.08). Bootstrapped coefficients for the three-predictor model were significant for age (CI -0.7993, -0.4940) and the control/styrene contrast (CI -7.479, -0.459). Based on the coefficient of the regression (β), styrene-exposed workers are expected to present DPOAE amplitudes around 4 dB SNR lower (i.e. worse) than control-group workers in the right and left ears. At 6 kHz for the left ear, predictors significantly improved prediction in steps 1 and 2 and group category generated a significant improvement in step 3 (*F =* 3.51, *p =* .0.03, *R*^2^ = 0.31, *f*^*2*^ = 0.46). Bootstrapped coefficients were significant for age (CI -0.8735, -0.5490) and the control/styrene contrast (CI -7.248, -1.062). For the right ear at 6 kHz, significant improvement was observed in steps 1 and 2, with trend-wise results when introducing group category in step 3 (*F =* 2.55, *p =* 0.07, *R*^2^ = 0.31, *f*^*2*^ = 0.44). Bootstrapped coefficients revealed significance for age (CI -0.7698, -0.5080) and the control/styrene contrast (CI -6.990, -0.480). Based on the coefficient of the regression (β), styrene-exposed workers are expected to present DPOAE amplitudes around 3.7 and 4.1 dB SNR lower (i.e. worse) than control-group workers in right and left ear, respectively.

**Table 3:** HLR results for distortion product otoacoustic emissions (1.5–6 kHz) for the right and left ears. Bootstrapped parameters (CI, bias and SE) are only reported when the increase in step 3 was found to be significant (< 0.05) or trend-wise (<0.1). All significant improvements in step 2 and/or step 3 are marked with **. Trend-wise improvements are marked with *.

**Right ear, 1.5 kHz**						
	β	P	R^*2*^	CI	bias	se
**Step 1**						
Age	-0.24	<0.001	0.07			
**Step 2**						
Age	-0.25	<0.001	0.06			
Mean lifetime noise exposure	0.03	0.60				
**Step 3**						
Age	-0.27	<0.001	0.06			
Mean lifetime noise exposure	-0.09	0.53				
Control/Styrene	-0.91	0.50				
Control/Noise	1.94	0.70				
**Left ear, 1.5 kHz**						
	β	P	R^*2*^	CI	bias	se
**Step 1**						
Age	-0.20	<0.001	0.05			
**Step 2**						
Age	-0.21	<0.001	0.04			
Mean lifetime noise exposure	0.06	0.35				
**Step 3**						
Age	-0.22	<0.001	0.04			
Mean lifetime noise exposure	0.03	0.79				
Control/Styrene	-0.36	0.79				
Control/Noise	0.25	0.09				
**Right ear, 2 kHz**						
	β	P	R^*2*^	CI	bias	se
**Step 1**						
Age	-0.22	<0.001	0.05			
**Step 2**						
Age	-0.23	<0.001	0.05			
Mean lifetime noise exposure	0.07	0.28				
**Step 3**						
Age	-0.24	<0.001	0.05			
Mean lifetime noise exposure	0.03	0.81				
Control/Styrene	-1.76	0.21				
Control/Noise	-0.08	0.97				
**Left ear, 2 kHz**						
	β	P	R^*2*^	CI	bias	se
**Step 1**						
Age	-0.21	<0.001	0.05			
**Step 2**						
Age	-0.21	<0.001	0.04			
Mean lifetime noise exposure	0.00	0.95				
**Step 3**						
Age	-0.20	<0.001	0.04			
Mean lifetime noise exposure	0.06	0.56				
Control/Styrene	-2.31	0.09				
Control/Noise	-2.53	0.36				
**Right ear, 3 kHz**						
	β	P	R^*2*^	CI	bias	se
**Step 1**						
Age	-0.49	<0.001	0.22			
**Step 2**						
Age	-0.48	<0.001	0.21			
Mean lifetime noise exposure	-0.07	0.23				
**Step 3**						
Age	-0.47	<0.001	0.21			
Mean lifetime noise exposure	0.02	0.85				
Control/Styrene	-2.17	0.12				
Control/Noise	-2.75	0.33				
**Left ear, 3 kHz**						
	β	P	R^*2*^	CI	bias	se
**Step 1**						
Age	-0.48	<0.001	0.22			
**Step 2**						
Age	-0.46	<0.001	0.23**			
Mean lifetime noise exposure	-0.13	0.03				
**Step 3**						
Age	-0.45	<0.001	0.23			
Mean lifetime noise exposure	-0.05	0.72				
Control/Styrene	-1.87	0.16				
Control/Noise	-2.28	0.40				
**Right ear, 4 kHz**						
	β	P	R^*2*^	CI	bias	se
**Step 1**						
Age	-0.52	<0.001	0.19			
**Step 2**						
Age	-0.50	<0.001	0.20**			
Mean lifetime noise exposure	-0.19	0.01				
**Step 3**						
Age	-0.49	<0.001	0.21			
Mean lifetime noise exposure	-0.12	0.47				
Control/Styrene	-3.29	0.04				
Control/Noise	-2.70	0.41				
**Left ear, 4 kHz**						
	β	P	R^*2*^	CI	bias	se
**Step 1**						
Age	-0.47	<0.001	0.16			
**Step 2**						
Age	-0.44	<0.001	0.18**			
Mean lifetime noise exposure	-0.20	0.006				
**Step 3**						
Age	-0.44	<0.001	0.18			
Mean lifetime noise exposure	-0.15	0.36				
Control/Styrene	-1.39	0.37				
Control/Noise	-1.44	0.65				
**Right ear, 5 kHz**						
	β	P	R^*2*^	CI	bias	se
**Step 1**						
Age	-0.69	<0.001	0.25			
**Step 2**						
Age	-0.66	<0.001	0.26**			
Mean lifetime noise exposure	-0.19	0.01				
**Step 3**						
Age	-0.65	<0.001	0.27*	-0.7993, -0.4940	0.000	0.07
Mean lifetime noise exposure	-0.01	0.93		-0.4188, 0.4046	0.001	0.21
Control/Styrene	-4.01	0.02		-7.479, -0.459	-0.020	1.77
Control/Noise	-5.07	0.16		-13.135, 2.784	-0.021	4.07
**Left ear, 5 kHz**						
	β	P	R^*2*^	CI	bias	se
**Step 1**						
Age	-0.60	<0.001	0.26			
**Step 2**						
Age	-0.57	<0.001	0.27**			
Mean lifetime noise exposure	-0.17	0.01				
**Step 3**						
Age	-0.57	<0.001	0.29**	-0.6884, -0.4575	0.0006	0.05
Mean lifetime noise exposure	-0.07	0.66		-0.4467, 0.2576	0.002	0.17
Control/Styrene	-4.12	0.006		-6.864, -0.833	-0.22	1.52
Control/Noise	-3.70	0.22		-9.876, 3.185	-0.04	3.43
**Right ear, 6 kHz**						
	β	P	R^*2*^	CI	bias	se
**Step 1**						
Age	-0.70	<0.001	0.27			
**Step 2**						
Age	-0.66	<0.001	0.30**			
Mean lifetime noise exposure	-0.26	<0.001				
**Step 3**						
Age	-0.65	<0.001	0.31*	-0.7698, -0.5080	-0.001	-0.07
Mean lifetime noise exposure	-0.15	0.38		-0.5232, 0.2335	-0.0003	0.19
Control/Styrene	-3.74	0.02		-6.990, -0.480	-0.017	1.62
Control/Noise	-3.57	0.29		-11.110, 3.649	0.006	3.76
**Left ear, 6 kHz**						
	β	P	R^*2*^	CI	bias	se
**Step 1**						
Age	-0.71	<0.001	0.28			
**Step 2**						
Age	-0.68	<0.001	0.30**			
Mean lifetime noise exposure	-0.20	0.01				
**Step 3**						
Age	-0.69	<0.001	0.31**	-0.8735, -0.5490	0.0009	0.07
Mean lifetime noise exposure	-0.18	0.31		-0.5221, 0.1748	0.0000	0.17
Control/Styrene	-4.11	0.01		-7.248, -1.062	-0.007	1.58
Control/Noise	-2.14	0.52		-9.084, 4.252	-0.02	3.33

β: Estimated coefficient of the exploratory variables that indicates changes in the auditory outcome. Note that coefficients are not standardised.

*Trend-wise (p>0.05)

**p<0.05

### Auditory brainstem response

Table 4 displays the mean, standard error and confidence interval, adjusted for age and mean lifetime noise exposure for the ABR components across the three groups (styrene-exposed, noise-exposed and control group subjects).

**Table 4:** Mean, standard error and 95% confidence interval adjusted by age and noise exposure levels for ABR results for all three groups of workers.

	Styrene-exposed group		Noise-exposed group		Non-exposed control group	
Variable	(n = 98)		(n = 111)		(n = 70)	
	Mean (S.E)	95%CI	Mean (S.E)	95% CI	Mean (S.E)	95%CI
**Wave I RE**	1.73 (.02)	1.69–1.78	1.70 (.03)	1.64–1.76	1.71 (.03)	1.65–1.77
**Wave III RE**	3.84 (.02)	3.80–3.88	3.88 (.02)	3.82–3.94	3.91 (.02)	3.85–3.96
**Wave V RE**	5.70 (.03)	5.64–5.76	5.69 (.04)	5.60–5.78	5.82 (.04)	5.74–5.91
**I-III IPL RE**	2.10 (.02)	2.05–2.15	2.18 (.03)	2.10–2.25	2.19 (.03)	2.12–2.27
**I-V IPL RE**	3.97(.03)	3.90–4.03	3.99 (.05)	3.89–4.08	4.11 (.05)	4.01–4.21
**III-V IPL RE**	1.86 (.02)	1.81–1.92	1.80 (.04)	1.73–1.88	1.91 (.04)	1.83–1.99
**Wave I LE**	1.72 (.01)	1.69–1.76	1.66 (.02)	1.61–1.71	1.73 (.02)	1.68–1.78
**Wave III LE**	3.84 (.02)	3.80–3.89	3.87 (.03)	3.81–3.93	3.91 (.03)	3.85–3.97
**Wave V LE**	5.69 (.03)	5.63–5.74	5.65 (.04)	5.56–5.73	5.81 (.04)	5.73–5.90
**I-III IPL LE**	2.12 (.02)	2.07–2.17	2.20 (.03)	2.13–2.28	2.17 (.03)	2.10–2.25
**I-V IPL LE**	3.96 (.03)	3.90–4.03	3.98 (.04)	3.88–4.08	4.08 (.04)	3.98–4.17
**III-V IPL LE**	1.84 (.02)	1.78–1.89	1.77 (.04)	1.69–1.85	1.90 (.04)	1.82–1.98

All values are in milliseconds

RE: right ear; LE: left ear

IPL: Inter-peak latency

S.E: Standard error

95%CI: 95% confidence interval

HLR analysis for ABR wave V latency in the right ear (see Table 5) revealed a significant contribution of age (step 1) and group category in step 3 (*F =* 7.01, *p =* 0.001). Values for the three-predictor model revealed a small effect (*R*^2^ = 0.09, *f*^*2*^ = 0.10). Bootstrapped coefficients showed significance for age (CI 0.0038, 0.0140) and the control/styrene contrast (CI -0.1966, -0.0371). Similar results were observed for the left ear (see Table 5), with model significance only observed in step 1 and step 3 (*F =* 5.49, *p =* 0.004, *R*^2^ = 0.11, *f*^*2*^ = 0.12). Bootstrapped coefficients were significant for age (CI 0.0059, 0.0122), the control/styrene contrast (CI -0.1963, -0.0585), and the control/noise contrast (CI -0.3225, -0.307). Based on the coefficient of the regression (β), styrene-exposed workers are expected to present wave V latency around 0.12 ms earlier than control-group workers in the right and left ears.

**Table 5:** HLR results for the auditory brainstem response wave V latency (ms) for the right and left ears. Bootstrapped parameters (CI, bias and SE) are only reported when the increase in step 3 was found to be significant (< 0.05) or trend-wise (<0.1). All significant improvements in step 2 and/or step 3 are marked with **. Trend-wise improvements are marked with *.

**Right ear**						
	β	p	R^*2*^	CI	bias	se
**Step 1**						
Age	0.007	<0.001	0.07			
**Step 2**						
Age	0.007	<0.001	0.06			
Mean lifetime noise exposure	0.0006	0.73				
**Step 3**						
Age	0.007	<0.001	0.09**	0.0038, 0.0140	0.00002	0.001
Mean lifetime noise exposure	0.005	0.20		-0.036, 0.0140	0.00004	0.004
Control/Styrene	-0.12	0.002		-0.1966, -0.0371	-0.0001	0.04
Control/Noise	-0.14	0.07		-0.3050, 0.0444	-0.0008	0.08
**Left ear**						
	β	p	R^*2*^	CI	bias	se
**Step 1**						
Age	0.008	<0.001	0.09			
**Step 2**						
Age	0.008	<0.001	0.09			
Mean lifetime noise exposure	-0.0003	0.84				
**Step 3**						
Age	0.009	<0.001	0.11**	0.0059, 0.0122	0.00001	0.001
Mean lifetime noise exposure	0.006	0.14		-0.0018, 0.0141	0.00002	0.004
Control/Styrene	-0.12	0.001		-0.1963, -0.0585	-0.0004	0.035
Control/Noise	-0.17	0.02		-0.3225, -0.307	-0.0006	0.07

β: Estimated coefficient of the exploratory variables that indicates changes in the auditory outcome. Note that coefficients are not standardised.

**p<0.05

**Fig 4:**
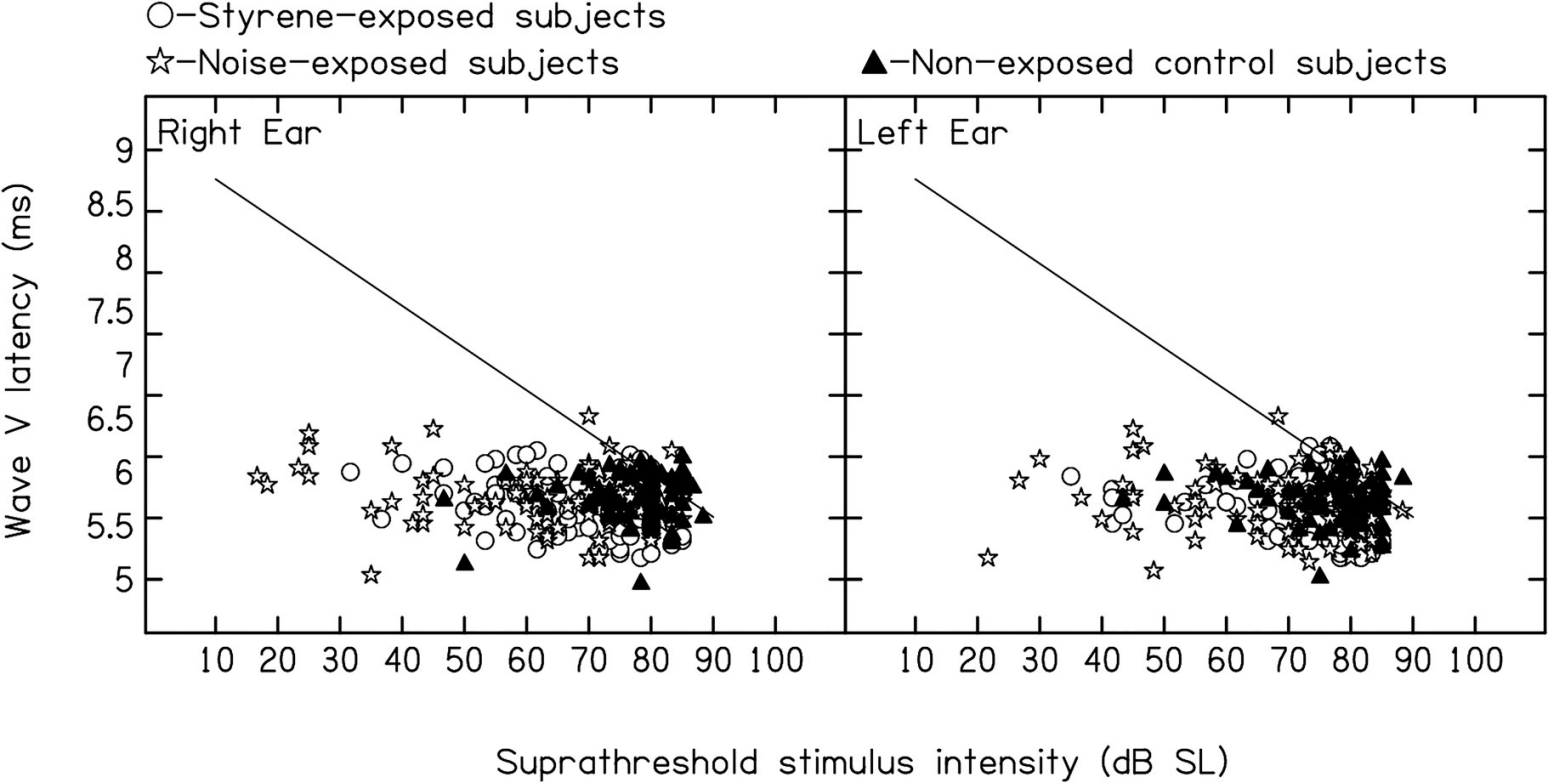
Delta ABR wave V values for each subject in each group for the right and left ears. The line represents the expected value for ABR wave V latency as a function of dB SL (Delta ABR wave V = 0).

**Delta ABR wave V**. Fig 4 displays the values for delta ABR wave V for each subject in each group of workers in the right and left ears.

HLR analysis (see Table 6) for delta ABR wave V showed a significant contribution of predictors in step 2 and step 3 (*F =* 6.36, *p =* 0.002, *R*^2^ = 0.07, *f*^*2*^ = 0.07) for the right ear. Bootstrapped coefficients were only significant for the control/styrene contrast (CI -0.3201, -0.0902). Based on the coefficient of the regression (β), styrene-exposed workers are expected to have wave V latency around 0.2 ms earlier than control-group workers at the same presentation level in dB SL of the stimuli in the right ear. Similar results were observed for the left ear, with group category improving prediction in step 3 (*F =* 3.31, *p =* 0.03, *R*^2^ = 0.02, *f*^*2*^ = 0.02). Significance was only found for the control/styrene contrast (CI -0.2452, -0.0360). Based on the coefficient of the regression (β), styrene-exposed workers are expected to have wave V latency around 0.14 ms earlier than control-group workers at the same presentation level in dB SL of the stimuli in the left ear.

**Table 6:** HLR results for the delta ABR wave V for the right and left ears. Bootstrapped parameters (CI, bias and SE) are only reported when the increase in step 3 was found to be significant (< 0.05) or trend-wise (<0.1). All significant improvements in step 2 and/or step 3 are marked with **. Trend-wise improvements are marked with *.

**Right ear**						
	β	p	R^*2*^	CI	bias	se
**Step 1**						
Age	0.0008	0.74	0.00			
**Step 2**						
Age	0.002	0.37	0.03**			
Mean lifetime noise exposure	-0.01	0.001				
**Step 3**						
Age	0.001	0.51	0.07**	-0.0032, 0.0050	0.00005	0.002
Mean lifetime noise exposure	-0.01	0.13		-0.0273, 0.0050	-0.0001	0.008
Control/Styrene	-0.20	0.001		-0.3201, -0.0902	0.001	0.58
Control/Noise	-0.08	0.54		-0.3608, 0.2205	0.003	0.14
**Left ear**						
	β	p	R^*2*^	CI	bias	se
**Step 1**						
Age	0.002	0.4	0.00			
**Step 2**						
Age	0.002	0.24	0.009			
Mean lifetime noise exposure	-0.005	0.05				
**Step 3**						
Age	0.002	0.31	0.02**	-0.0021, 0.0073	0.00002	0.002
Mean lifetime noise exposure	-0.004	0.44		-0.0.191, 0.0096	0.0001	0.007
Control/Styrene	-0.14	0.01		-0.2452, -0.0360	0.0002	0.05
Control/Noise	-0.06	0.56		-0.3257, 0.1907	0.001	0.13

β: Estimated coefficient of the exploratory variables that indicates changes in the auditory outcome. Note that coefficients are not standardised.

**p<0.05

## Discussion

### Pure-tone thresholds

The regression models showed an effect of age (step 1) and mean lifetime noise exposure (step 2) on pure-tone thresholds (1–8 kHz) in the right and left ears. In addition, a modest effect of styrene exposure, controlled for age and mean lifetime noise exposure (step 3), was observed on hearing thresholds (1–8 kHz) in both ears. Note that these interpretations are mainly based on p-values (when comparing steps 2 and 3 of HLR) rather than R^2^ contribution of styrene. The possible association between styrene exposure and hearing thresholds agrees with previous research investigating subjects exclusively exposed to styrene. Morata et al. [15], found worse hearing thresholds for the range 2–6 kHz in workers (n = 65) exposed to very low concentrations of styrene (16 mg/m3) compared to non-exposed subjects. We found similar results for a wide range of audiometric frequencies in our previous study [16]. These two studies and the present study contrast with the findings by Muijser et al. [11]. The latter authors found no significant differences for hearing thresholds between styrene-exposed subjects from the glass-reinforced plastic industry and control-group subjects without styrene exposure. However, the control group selected by Muijser et al. [11] was exposed to low levels of noise and this was not accounted for in the statistical analysis. However, a statistically significant difference in hearing thresholds was found between workers with high exposure to styrene (at a mean concentration of 138 mg/m^3^) and workers with low exposure to styrene (at 61 mg/m^3^). Further research should be conducted with the aim to determine an association between concentrations of styrene exposure over time and changes in hearing thresholds controlling for noise exposure levels.

Regarding the ultra-high frequency thresholds, Pearson’s residuals for the Chi-Square test showed that a measurable threshold at 16 kHz was significantly present in control group participants as compared to noise-exposed and styrene-exposed participants. In addition, noise-exposed participants showed a significantly low presence of a measurable hearing threshold at 16 kHz in both ears as compared to the other two groups. Therefore, we conclude that ultra-high frequency is not sensitive enough to detect the adverse auditory effects of styrene exposure, at least at the exposure levels of this sample of workers. Morioka et al. [17] found that subjects exposed to solvents including styrene for 5 years or more presented reduced upper-level hearing in comparison with non-exposed control subjects. In addition, this reduction was dose-dependent and was related to airborne concentrations of styrene and mandelic acid concentrations in urine. However, the procedures used in this study differed from the procedures used by Morioka et al [17]. In this study we tested only 12 and 16 kHz with the aim to determine whether or not a measurable threshold was obtained at each frequency. Morioka et al [17]. tested the entire range of ultra-high frequency and determined the highest frequency at which the person could hear. Further research should be conducted with the aim to investigate the effects of styrene exposure on the upper level of hearing in samples of workers exposed to different levels of styrene.

### Otoacoustic emissions

Age was significantly associated with DPOAE amplitudes at all frequencies (1.5–6 kHz) in both ears. This result was expected, as it has been extensively documented that DPOAE amplitudes decrease with age (e.g. [33,34,35]). Mean lifetime noise exposure was also significantly associated with DPOAE amplitudes in both ears. Specifically, this association was observed for the frequency range 3–6 kHz. Noise exposure has been shown to have an adverse effect on DPOAE amplitudes at frequency ranges similar to the one observed in this study [36,37]. When the previous two variables (i.e., age and mean lifetime noise exposure) were controlled for in the regression model (step 3), group category was modestly, yet significantly associated with DPOAE amplitudes at 5 and 6 kHz in the right and left ears. Specifically, styrene-exposed subjects showed significantly lower (i.e., poorer) DPOAE amplitudes than control-group subjects. As previously mentioned, such an association was based on p-values rather than R^2^ contribution of styrene. Note that the effect of the styrene group on DPOAE (step 3) is rather modest. Based on R^2^ contributions, noise exposure level (step 2) has a stronger effect on DPOAE than styrene exposure. The latter taken as a categorical variable. In addition, styrene exposure seems to be associated with a narrower range of frequencies (5–6 kHz) than noise exposure (3–6 kHz). Based on these results, we suggest that styrene exposure is associated, at least partially, with OHC dysfunction at high frequencies (5–6 kHz). Also, this finding suggests that the effect of styrene exposure on sound detection abilities is likely to be induced, at least in part, by cochlear dysfunction (i.e., OHC). This possible finding about styrene-induced cochlear dysfunction is in agreement with previous animal studies showing that at higher styrene exposure levels than in the present study, styrene adversely affects the OHC in rats [10,38,39]. Similarly, Sisto et al. [40] found a significant negative correlation between DPOAE amplitudes and the concentration of styrene metabolites in urine in human subjects. DPOAEs were able to discriminate between exposed workers and the controls. No other studies have been identified in the literature showing an adverse effect of styrene exposure on otoacoustic emission amplitudes in human subjects.

### Auditory brainstem response

Age and styrene exposure were significantly associated with ABR wave V latency in both ears. Older ages were associated with longer latencies, whereas styrene exposure was associated with shorter latencies. In addition, for the left ear, noise-exposed subjects presented with significantly shorter wave V latency than did control-group subjects. Note that the combined effect of age, styrene exposure and noise exposure on wave V latency was rather small. Age and styrene exposure explained 9% of the variance of wave V latency in the right ear. Age, styrene exposure and noise exposure explained 11% of the variance of wave V latency in the left ear. The effect of age on ABR wave V latency agrees with previous studies showing that wave V latency increases with age (e.g. [41–43]). We believe that the effect of styrene and noise on ABR results (i.e., shorter latencies) relates to an adverse cochlear effect induced by these agents. Further discussion of this is provided below.

Prosser and Arslan [31] suggested that cochlear dysfunction can be differentiated from retrocochlear dysfunction based on the absolute ABR wave V latency obtained at a particular intensity in dB SL. For this, the absolute ABR wave V latency must be compared with the expected latency obtained in the normal-hearing population at the same intensity in dB SL [31]. For example, if someone has a pure tone average (4–6 kHz) of 40 dB HL, presenting a click stimulus at 90 dB nHL (50 dB SL) should produce a wave V latency of 6.98 ms (based on normative data; [31]). For this particular example, latencies below (shorter) this value (i.e., 6.98 ms) suggest cochlear dysfunction and latencies above (longer) this value suggest retrocochlear dysfunction. Thus, the authors pointed out that the absolute ABR wave V latency in persons with cochlear dysfunction would shift to lower levels (shorter latencies) than in normal-hearing individuals. The authors suggested that in persons with retrocochlear dysfunction, the absolute ABR wave V latency would shift to higher levels (longer latencies) than in normal-hearing individuals. For this, Prosser and Arslan [31] suggested a formula to calculate a person’s shift from the latency obtained in normal-hearing subjects. Numbers below the expected value indicate shorter latencies and numbers above the expected value indicate longer latencies compared to the normative data. For each individual from all three groups, we calculated for both ears the observed shift of wave V latency in comparison to the normative data presented by Prosser and Arslan [31]. We plotted the latency of wave V obtained in each subject and the corresponding suprathreshold intensity (dB SL) used for stimulus presentation, as well as the predicted line (see Fig 4) for the latency of wave V at each intensity (in dB SL). Fig 4 shows that non-exposed control subjects obtained values closer to the predicted line compared to styrene-exposed and noise-exposed subjects. Also, a high proportion of styrene-exposed and noise-exposed subjects obtained values below the predicted line and thus had shorter latencies than expected. As mentioned above, Prosser and Arslan [31] suggested that values below the predicted line correspond to individuals with cochlear dysfunction. Therefore, we conclude that the obtained ABR data suggest that styrene is associated with cochlear dysfunction. This finding is supported by the data obtained with DPOAE.

### Limitations of the study and suggestions for further research

A major fact to consider is the high percentage of workers overexposed to styrene (94%) based on the Polish OEL. Note, however, that the OEL for styrene in Poland is one of the lowest in the world. Thus, applying styrene OELs from other countries such as the US would lower the percentage of overexposed workers. The major problem with this high percentage of overexposed workers is that these results cannot be extrapolated to populations of workers who have been exposed to permissible levels of styrene in Poland. Therefore, we suggest that further research should be conducted on workers exposed to styrene levels within OELs. This would help determine whether the results found in this study are replicable in such a sample of workers. Another point is the co-exposure to acetone and dichloromethane among styrene-exposed subjects. Pryor [44] categorised both agents as non-ototoxic. Note that there is a report of a woman who developed bilateral hearing loss after overexposure to dichloromethane [45]. Thus, we hypothesise that the auditory signs observed in styrene-exposed subjects may not be exclusive to styrene exposure. Acetone and dichloromethane may have at least modified the metabolism of styrene, resulting in a more severe effect on the auditory system than styrene exposure alone. Further studies should be carried out to test this hypothesis.

In this study, we did not find signs of central auditory dysfunction, as measured with the ABR. However, we cannot conclude that styrene does not affect the central auditory nervous system. Some studies have shown that mixtures of solvents are associated with central auditory dysfunction in humans (e.g. [46,47]). In addition, in a previous study, we found that styrene exposure was associated with temporal processing difficulties [21]. Temporal processing is one of the functions associated with the central auditory nervous system [48]. The present study did not involve investigating temporal processing or other psychoacoustical tasks associated with the central auditory system. Further studies should include both psychoacoustical and electrophysiological techniques to evaluate the central auditory system in styrene-exposed subjects.

## Conclusion

The results of the DPOAE and ABR procedures carried out in styrene-exposed, noise-exposed and non-exposed subjects suggest that styrene exposure is likely to be associated with cochlear dysfunction in humans. We therefore hypothesise that the poorer hearing thresholds observed in styrene-exposed subjects compared to non-exposed subjects are due, at least in part, to styrene-induced ototoxicity. We conclude that because of the differences in DPOAEs between groups, this procedure can be used to monitor hearing along with pure-tone audiometry in styrene-exposed workers. However, due to the small effect of styrene exposure on the auditory outcomes investigated in this study, further research should be conducted with the aim to determine possible dose-response relationships between styrene exposure levels, controlling for noise exposure, and auditory outcomes.

## Supporting information

S1 File Data set. (XLS)
